# Printed PEDOT:PSS Trilayer: Mechanism Evaluation and Application in Energy Storage

**DOI:** 10.3390/ma13020491

**Published:** 2020-01-20

**Authors:** Inga Põldsalu, Kätlin Rohtlaid, Cedric Plesse, Frédéric Vidal, Ngoc Tuan Nguyen, Anna-Liisa Peikolainen, Tarmo Tamm, Rudolf Kiefer

**Affiliations:** 1 Intelligent Materials and Systems Lab, Institute of Technology, University of Tartu, Nooruse 1, 50411 Tartu, Estonia; inga.p6ldsalu@gmail.com (I.P.);anna.liisa.peikolainen@ut.ee (A.-L.P.); tarmo.tamm@ut.ee (T.T.); 2 Centre for Molecular Medicine Norway, Faculty of Medicine, University of Oslo, 0318 Oslo, Norway; 3 Laboratoire de Physicochimie des Polymères et des Interfaces, Université de Cergy-Pontoise, 15 mail Gay Lussac, 95031 Cergy-Pontoise, France; katlin.rohtlaid@gmail.com (K.R.); cedric.plesse@u-cergy.fr (C.P.); frederic.vidal@u-cergy.fr (F.V.); 4 Faculty of Applied Sciences, Ton Duc Thang University, Ho Chi Minh City 700000, Vietnam; nguyenngoctuan@tdtu.edu.vn

**Keywords:** PEDOT:PSS-IPN trilayer, ink-jet printing, linear actuation, three different electrolytes, energy storage

## Abstract

Combining ink-jet printing and one of the most stable electroactive materials, PEDOT:PSS (poly(3,4-ethylenedioxythiophene):poly(styrene sulfonate)), is envisaged to pave the way for the mass production of soft electroactive materials. Despite its being a well-known electroactive material, widespread application of PEDOT:PSS also requires good understanding of its response. However, agreement on the interpretation of the material’s activities, notably regarding actuation, is not unanimous. Our goal in this work is to study the behavior of trilayers with PEDOT:PSS electrodes printed on either side of a semi-interpenetrated polymer network membrane in propylene carbonate solutions of three different electrolytes, and to compare their electroactive, actuation, and energy storage behavior. The balance of apparent faradaic and non-faradaic processes in each case is discussed. The results show that the primarily cation-dominated response of the trilayers in the three electrolytes is actually remarkably different, with some rather uncommon outcomes. The different balance of the apparent charging mechanisms makes it possible to clearly select one electrolyte for potential actuation and another for energy storage application scenarios.

## 1. Introduction

The demand for small smart devices in modern, fast-developing technologies requires materials that can be adapted on flexible substrates for applications such as flexible electrodes in batteries [1], super capacitors [2] and other energy storage devices [3,4], flexible solar cells [5], micro-actuators [6], and sensors [7,8]. Conducting polymers may be suitable for those devices, but their formation as simple, chemically-deposited coatings leads to certain limitations of the materials chosen; furthermore, their performance is reduced in comparison to electrochemically-polymerized conducting polymers. PEDOT:PSS solutions can be printed on flexible fabrics [9], with applications in solar cells [10], biomedical sensors [11], electrochromic devices [12], soft actuators [13,14], and capacitors [9]. Actuators fabricated by printing PEDOT:PSS on NBR/PEO fabrics [15] have shown moderate strain in linear and bending actuation. It remains unclear if the underlying mechanism is mainly faradaic [16] or non-faradaic [17], in cases involving the use of ionic liquids as the electrolyte, claims have been made that the electrical double layer (non-faradaic) processes dominated [18]. Conducting polymers, even chemically-formed ones [19], typically follow faradaic process mechanisms where the charge determines the actuation response during redox reactions. The conducting polymer is oxidized at a given applied potential, resulting in a delocalized charge, whereas (solvated) counter-ions balance the charge, leading to swelling. During reduction, the charge is removed and the excess ions leave the polymer film, leading to film contraction. Under the assumption that PEDOT:PSS follows a faradaic mechanism, the charge of PEDOT in the oxidized state is already balanced by immobile PSS^−^ anions, while during reduction, the excess negative charge of PSS^−^ leads to the ingress of (solvated) cations [15] with expansion upon reduction (discharging). 

On the other hand, the highly porous PEDOT:PSS network has a huge surface area, the charging of which leads to the formation of an electric double layer (EDL). The alterations in EDL upon charging/discharging are known as the non-faradaic [20,21] mechanism of energy storage of carbon-based supercapacitors, as well as actuation. Our goal in this work was to shed some light on the dominant processes of the PEDOT:PSS (PP) printed on both sides of nitrile butadiene rubber/polyethylene oxide (NBR/PEO), a semi-interpenetrating network (IPN) forming a PP–IPN trilayer. Different electrolytes were applied in a propylene carbonate solution, attempting to relate the nature of the electrolyte to the electroactivity mechanism.

First, 1-ethyl-2,3-dimethylimidazolium trifluoromethanesulfonate (EDMICF_3_SO_3_), lithium bis(trifluoromethane)sulfonimide (LiTFSI), and sodium perchlorate (NaClO_4_) in propylene carbonate were used to study and compare the linear actuation response in strain and stress (blocking force) of PP–IPN trilayers. Electrochemical, microscopy, and elemental analysis tools were applied for the study. 

## 2. Material and Methods

### 2.1. Formation of the IPN and Assembly of the PP–IPN

The semi-IPN [22] consisted of nitrile butadiene rubber (NBR, Lanxess, (Köln, Germany), providing flexibility and a poly (ethylene oxide) (PEO) network providing ionic conductivity in a 50:50 wt.% ratio. NBR (17 wt.%) was dissolved in cyclohexanone (Aldrich, >99.8%), stirred overnight until fully dissolved. PEO (50 wt.% vs. NBR) was comprised of poly(ethylene glycol) dimethacrylate (PEGDM, Aldrich, M_n_ = 750 g mol^−1^) (25 wt.% of PEO network) and poly(ethylene glycol) methacrylate methyl ether (PEGM, Aldrich, M_n_ = 500 g mol^−1^) (75 wt.% of PEO network). To obtain the polymerization solution for semi-IPN, the PEO was combined with the NBR solution and stirred for of 30 min. The initiator, dicyclohexylperoxidicarbonate (DCPD, Group Arnaud) (3 wt.% of PEO network), was included in the solution to start the polymerization process with continuous stirring. The solution was then degassed. The assembly of the PP–IPN layer was processed similarly to previous research [15]. Figure S1 shows the principle scheme of the PP–IPN trilayer assembly and a description of it. The printed PEDOT:PSS on both sides of the IPN membrane formed the PP–IPN trilayer, with a total thickness of 13 ± 1 µm. 

### 2.2. Characterization of PP–IPN

The cross-section of the PP–IPN trilayer was investigated with scanning electron microscopy (SEM, Helios NanoLab 600, FEI, Oregon, USA) to assure uniform electrode thickness on both sides. Energy dispersive X-ray (EDX) spectrometry (Wiesbaden, Germany) of the cross sections was performed to determine the distribution of sulfur, oxygen, and carbon. The sheet conductivity *σ_S_* of the electrodes of thickness *h* was determined according to Equation (1), by applying the four-point probe method with a surface resistivity meter (Guardian SRM) to measure the sheet resistance *R_s_*.

(1) $$\sigma_{S}=\frac{1}{R_{s\;}\times h\;}$$

### 2.3. Electroactivity of the Trilayers

The PP–IPN were cut into 1 cm × 0.1 cm strips (for each applied electrolyte, more than three samples were measured) and fixed at one end between two gold electrodes (working electrode) at the holder, and at the other end to the force sensor (TRI202PAD, Panlab, Barcelona, Spain). The free film length between the force sensor and the clamp was set to 1 mm. The samples were prestretched in the range of 1 µm (1%) for 4 h before the measurements commenced. The self-made electro–chemo–mechanical deformation (ECMD) device [23] includes a linear muscle analyzer and a potentiostat (Biologic PG581, Göttingen, Germany). Before length change measurements, the elastic moduli of the PP–IPN trilayers were measured with a 1 μm length change. The strain ε (%) = (L_0_ − L_1_/L_0_) × 100 (isotonic ECMD—constant force of 4.9 mN) was calculated from the length change of the films. L_0_ is the original length of the PP–IPN trilayer, and L_1_ the current length of the trilayer. For isometric ECMD (constant length of 1 mm), the change of mass was calculated to stress σ (kPa) = mass change × g (gravity constant)/(width of the film × thickness) of the samples. The thickness of the PP–IPN trilayers was measured with a screw gauge (Eisco Labs, Rochester, NY, USA) in the electrolyte PC solutions (24 h), and again after actuation cycles.

The three electrode measurement cell (counter electrode a platinum sheet, an Ag/AgCl (3M KCl) reference electrode and the trilayer as a working electrode fixed onto a solid arm with gold contacts and the force sensor) contained the electrolyte solution. The propylene carbonate solutions of 1-ethyl-2,3-dimethylimidazolium trifluoromethanesulfonate (EDMICF_3_SO_3_, 99%, Sigma Aldrich, Saint-Quentin-Fallavier, France), lithium bis(trifluoromethane)sulfonimide (LiTFSI, 99.95%, Sigma Aldrich, Taufkirchen, Germany), and sodium perchlorate (NaClO_4_, > 99%, Sigma Aldrich, Taufkirchen, Germany) in the same concentration, i.e., 0.2 M, were used. At a potential range of 1.0 V to −0.6 V, cyclic voltammetry measurements (scan rate 5 mV s^−1^) were performed. Chronoamperometry in a frequency range of 0.0025 Hz to 0.1 Hz of the PP–IPN trilayers was conducted to obtain the diffusion coefficients from the samples by applying Equations (2) and (3) [24].

(2) $$\ln\left[1-\frac{Q}{Q_{t}}\right]=-bt$$

(3) $$D=\;\frac{b\times h^{2}}{2}$$

where *Q* is the charge density at each time period divided by the total charge density *Q_t_* at each applied frequency. The charge densities were obtained by the integration of the current density-time curves at each applied frequency. The term ln [1 − *Q*/*Q_t_*] obtained from Equation (2) against time t gives a curve from which the slope b can be obtained [24]. The thickness h of the PP–IPN trilayer with the slope b following Equation (3) gives us the diffusion coefficients at oxidation and reduction. 

To determine the specific capacitance, square wave current step measurements at frequencies ranging from 0.0025 to 0.1 Hz were performed, keeping a constant charge of 5 mC, with a current between ± 0.025 to ±1 mA. The slope at discharging (after IR drop) of the potential-time curves and the mass m (g) of the PP–IPN trilayer samples allowed us to calculate the specific capacitance, *C_s_*, using Equation (4) [20].

(4) $$C_{s}=\frac{i}{\left(-slope\times m\right)}$$

## 3. Results and Discussions

The adhesion of the electroactive layers to the membrane, as well as the mechanical properties of the membrane, play a key role in any soft electroactive device, in order to ensure stable and controlled performance. Too great a stiffness of the membrane, as shown previously for the conventional PVdF [25], will decrease the overall actuation response and limit the adhesion between the hydrophobic PVdF membrane and the printed (hydrophilic) PEDOT:PSS, which can lead to delamination. Here, we want to study the suitability of PP–IPN trilayers while attempting to shed some light on the actuation mechanism as a faradaic [26], non-faradaic [27], or mixed processes [15].

### 3.1. Characterization of PP–IPN Trilayers

**Figure 1:**
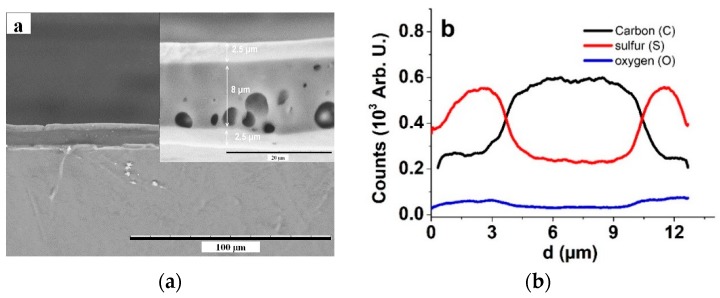
SEM images of a cross section of PP–IPN trilayer (scale bar 100 µm), including an inset for the thickness estimation of different layers. (**b**) EDX spectroscopy line scan of elemental composition of the cross section (inset of **a**) showing carbon (C, black), sulfur (S, red), and oxygen (O, blue) relative content.

Figure 1a shows a cross section image of the PP–IPN trilayer for the thickness estimation of the IPN and PEDOT:PSS coatings. 

The SEM image in Figure 1a clearly shows PEDOT:PSS layers on both sides of the IPN membrane. The inset of Figure 1a gives the thickness estimation of the membrane in the range of 8.0 ± 0.7 µm, and that of the printed PEDOT:PSS layers in the range of 2.5 ± 0.3 µm. The pores of the membrane seen in the inset of Figure 1a are the result of residual air bubbles, which did not show negative effects on the IPN stability or behavior. The EDX line scan spectra of the cross-section showed that the layer composition is significantly different, with moderately sharp interfaces. The carbon content was largest in the membrane, while the sulfur from both PEDOT and PSS was mostly seen in the electrode layers. The oxygen content was slightly higher in the electrode layer as well, due to the epoxy bridge in PEDOT and the PSS polymeric counter ions. 

The mass of dry PP–IPN at a dimension of 1.0 cm × 0.1 cm was in range of 72 ± 0.6 µg. The electronic conductivity of the PP–IPN trilayers after formation and drying was found to be in the range of 180 ± 16 S cm^−1^, i.e., slightly lower than some have reported [5]. The electronic conductivities of wet PP–IPN before (the trilayer was stored overnight in the electrolytes) and after actuation (200 charging/discharging cycles) of the applied electrolytes are shown in Table 1.

The results in Table 1 show that the electrolyte had a clear effect on the electronic conductivity before and after actuation, as well as on the swelling rate of the PP–IPN trilayers. The highest conductivity of EDMICF_3_SO_3_ was nearly double of that of the lowest electrolyte, i.e., NaClO_4_. The increase in conductivity in EDMICF_3_SO_3_ and LiTFSI after actuation cycles in PC has been reported before [28,29]. The conductivity of PP–IPN in the NaClO_4_ solution showed different behavior, i.e., a decrease in conductivity after actuation, accompanied by the lowest degree of swelling of the trilayers. 

The elastic modulus of each PP–IPN trilayer in different electrolytes was determined before and after the actuation cycles, as shown in Table 2.

In general, the elastic moduli were increased after the actuation cycles. Previously [30], it was found that changing the elastic modulus of the conducting polymer films has an important impact on the actuation. The smallest increase in the elastic modulus was found for PP–IPN operating in LiTFSI, with a 2.8-fold increase after actuation, followed for EDMICF_3_SO_3_, with a 3.7-fold increase, while the highest change was shown by NaClO_4_, i.e., a 4.8-fold increase.

### 3.2. Isotonic and Isometric ECMD Measurements of PP–IPN Trilayers

By analyzing the electroactivity response of the PP–IPN trilayers to a set of different electrochemical signals at various driving frequencies, the different thermodynamic and kinetic aspects of the underlying processes can be determined.

### 3.3. Cyclic Voltammetry Response

**Figure 2:**
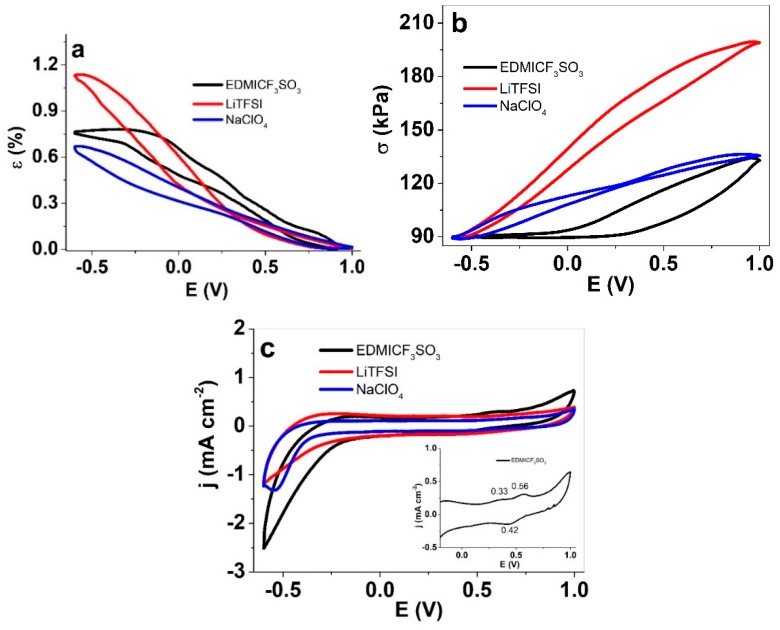
Isometric and isotonic ECMD measurements under cyclic voltammetry (1.0 to −0.6 V, 5 mV s^−1^, 5th cycle) of PP–IPN trilayers in PC solutions of: EDMICF_3_SO_3_ (black), LiTFSI (red) and NaClO_4_ (blue). Response as in (**a**) strain ε; (**b**) stress σ; (**c**) current density j against potential E (vs. Ag/AgCl (3M KCl)) with an inset for EDMICF_3_SO_3_ oxidation/reduction peaks.

Linear actuation measurements (isotonic and isometric ECMD) driven by cyclic voltammetry (scan rate 5 mV s^−1^) were performed in three different electrolytes in propylene carbonate. The results are presented in Figure 2.

While perhaps rather similar at first glance, the strain and stress responses have some remarkable differences. Quantitatively, both the maximum strain and stress differences were highest with LiTFSI as the electrolyte, 1.14% and 110 kPa, respectively. The lowest values, i.e., 0.67% and 47 kPa, were obtained in a NaClO_4_ solution. One aspect to consider is the changes in the elastic moduli compared before and after actuation (Table 2). The PP–IPN trilayers showed cation-dominated activity, resulting in expansion upon reduction in all the electrolytes. The shape of the curves, however, is somewhat different. Upon oxidation, the stress in EDMICF_3_SO_3_ remained low until well beyond 0 V potential, whereas for the other two electrolytes, the stress started to increase immediately from the beginning of the sweep. On the other hand, the strain of EDMICF_3_SO_3_ reached a plateau upon reduction a few hundred mV before the end of the sweep, while in the other two electrolytes, it continued to increase until the end. Such behavior could indicate higher mobility of the EDMI^+^ cations, meaning they have less (or virtually no) interaction with the polymer matrix during their flux. The current density curves (Figure 2c) also agree with this assumption, as the onset of the cation-flux coupled peaks [31] was towards the less negative potentials, and the current density was highest. One can also observe oxidation waves in the case of EDMICF_3_SO_3_ at 0.56 V, coupled with a reduction wave at 0.42 V (inset in Figure 2c). The reason why no features can be seen on the oxidation scan in the case of other electrolytes is that owing to the lower conductivity, the voltage on the terminals was not effectively transferred to the “bulk” of the electrodes.

The cation-dominated activity can be explained by the negatively-charged polyelectrolyte (PSS) in the PEDOT:PSS layer, which compensates for the positive charge of PEDOT upon oxidation. Upon reduction, the positive charge of PEDOT gets reduced and the PSS negative charge becomes balanced by the ingress of solvated cations [18]. We have three different cations in this study from the three different electrolytes (EDMICF_3_SO_3_, LiTFSI, and NaClO_4_), i.e., EDMI^+^, Li^+^ and Na^+^, which lead to different degrees of swelling of PEDOT:PSS upon reduction. The swelling rates depend on different factors, including the solvation number in propylene carbonate. The highest solvation number in PC is that of Li^+^ ions, i.e., 4–5 PC molecules [32], followed by Na^+^ ions with 3 PC molecules [33]. In the case of EDMI^+^ ions, the solvation number in PC is not available, but we assume that the delocalized charge on the imidazolium ring establishes a lower solvation number in PC.

The peak current density values for the EDMICF_3_SO_3_ electrolyte were 1.4 times higher than those of LiTFSI, and 2 times higher than those of NaClO_4_. Among other factors, this result is likely related to the higher conductivity of PP–IPN in EDMICF_3_SO_3_ (Table 1). The charge densities (not shown here) of PP–IPN operated in EDMICF_3_SO_3_, LiTFSI, and NaClO_4_ were 110 mC cm^−2^, 79 mC cm^−2^, and 57 mC cm^-1^, respectively.

### 3.4. Square Wave Potential Step Response

**Figure 3:**
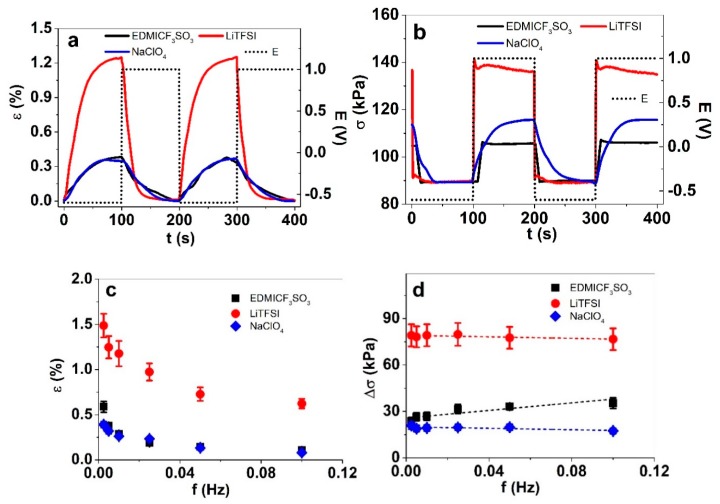
Square wave potential step (1.0 to −0.6 V, 0.005 Hz, 3rd and 4th cycle) response of PP–IPN trilayers in EDMICF_3_SO_3_ (■, black), LiTFSI (●, red) and NaClO_4_ (♦, blue) of (**a**) strain vs. time; (**b**) stress vs. time, with potential E (dotted); (**c**) strain vs. frequency; (**d**) the stress difference Δσ vs. frequency. The dotted lines in d show the linear fit, for orientation only.

Compared to the sweeping nature of cyclic voltammetry, square wave potential steps introduce abrupt jumps from one potential state to the other, bringing about a different kinetic response. Isometric and isotonic ECMD measurements of PP–IPN under square wave potential steps at 0.005 Hz were carried out. The response in strain and stress (blocking force) of the two subsequent cycles are shown in Figure 3a,b. The strain and stress difference against the applied frequency is shown in Figure 3c,d.

Figure 3a,b show the response profile of strain and stress measurements of the PP–IPN trilayer at 0.005 Hz in the three different electrolytes. As seen in the CV results, LiTFSI showed the highest strain and stress, followed by the relatively similar strains of the other two, and a clear second place in stress for NaClO_4_. A square wave potential step response of stress of the PP–IPN trilayer in LiTFSI and EDMICF_3_SO_3_ electrolytes at frequencies 0.0025–0.1 Hz is plotted in Figure S2.

The strain–frequency dependencies (Figure 3c) in all of the three electrolytes follow a very similar pattern: the highest values are reached at the lowest frequency, as expected. The difference in stress Δσ (stress at oxidation and reduction, Figure 3d), on the other hand, shows rather unusual behavior. Firstly, the stress of PP–IPN trilayers was only very slightly dependent on the applied frequency in LiTFSI and NaClO_4_. Secondly, the stress in EDMICF_3_SO_3_ actually increased slightly with increasing frequency. A slight fallback in strain in the case of a relatively low frequency actuation of PEDOT has been observed before [34]; this was assigned to a lower electro–chemo–mechanical coupling (compared to polypyrrole), allowing more solvent to accompany the ions upon ingress. Since the packing of a conducting polymer depends on the initial doping anion, the polymeric PSS, in our case, is expected to lead to an even more open structure, allowing more space in which the ions may move around. The latter is especially significant for the poorly-solvated EDMI^+^ cations, while Li^+^ naturally brings in the most solvent, leading to partial relaxation.

**Figure 4:**
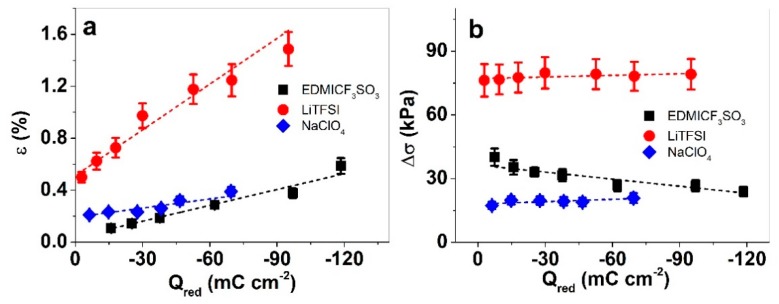
Square wave potential steps (1.0 V to −0.6 V) response of PP–IPN trilayer in EDMICF_3_SO_3_ (■, black), LiTFSI (●, red) and NaClO_4_ (♦, blue) in propylene carbonate. The charge densities at reduction (Figure S3b) were obtained at different applied frequencies by integrating the current density/time curves (Figure S3a, 0.005 Hz). The strain and stress differences against the charge density are shown in (**a**,**b**), respectively. The dashed lines are the linear fits, shown for orientation.

The strain and stress plotted against the charge density allowed us to quantify the electro–chemo–mechanical coupling or charge efficiency of each system; the results are presented in Figure 4a,b.

As could be expected from the current densities, the highest charge densities of the trilayers were those in EDMICF_3_SO_3_ at 0.0025 Hz with −118.5 ± 8.3 mC cm^−2^. Figure 4a shows that the strains increased with increasing charge density (on reduction) for all the applied electrolytes, thereby following the laws of faradaic processes [35], i.e., where charge density determines actuation response. The stress difference plots (Figure 4b) are, again, more interesting, with only a slight increase of stress difference with increasing charge density for PP–IPN trilayers operated in LiTFSI and NaClO_4_. In the case of the EDMICF_3_SO_3_ electrolyte, there was, in contrast, a small decrease of stress difference with increasing charge density. This discrepancy is likely related to the variation in the balance of the Faradaic and (apparent) EDL mechanisms of activity. As the conductivity of the trilayers in the three electrolyte solutions is remarkably different, so is the actual voltage driving the processes in the “bulk” of the electrodes; therefore, the higher-conductivity of EDMICF_3_SO_3_ makes it possible to reach voltages of more Faradaic-nature processes, while the lower conductivity electrolytes limit the processes to the EDL region.

**Figure 5:**
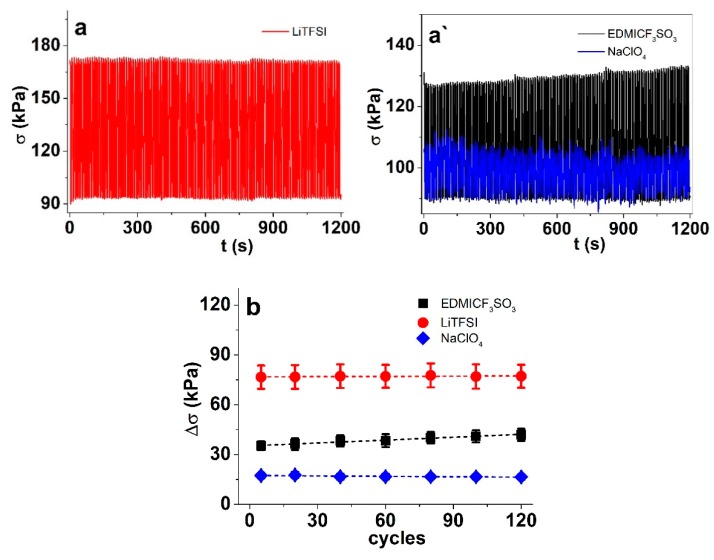
Stress response stability during square wave potential steps (0.1 Hz) for PP–IPN layers in EDMICF_3_SO_3_ (black, ■), LiTFSI (red, ●) and NaClO_4_ (blue, ♦) in (**a**,**a`)**: stress (blocking force) vs. time; (**b**) stress difference vs. cycle number.

Commonly, the electroactivity response of conducting polymer materials strongly depends on the history of the material (including the number of cycles, in cases of cycling). Figure 5 shows the stress response (blocking force) of the PP–IPN trilayer as a function of time and cycle count.

While the response was rather stable in all three electrolytes, LiTFSI stood out as the most stable (and with the highest stress, in both absolute values and in terms of the stress difference between the oxidized and reduced states). There was a slight decrease in stress for trilayers in NaClO_4_, while those in EDMICF_3_SO_3_ showed (somewhat surprisingly) an increase in the stress difference Δσ from 35 kPa at cycle 5 to 41.8 kPa at cycle 120. As the charge densities (Figure S4) during the response stability test stayed stable or slightly increased in time for all three electrolytes, the actual electroactivity was not lost, and there was no deterioration of the material. Instead, the electro–chemo–mechanical coupling was lowered, as the flux of (solvated) cations gullied their way into the material over time.

**Figure 6:**
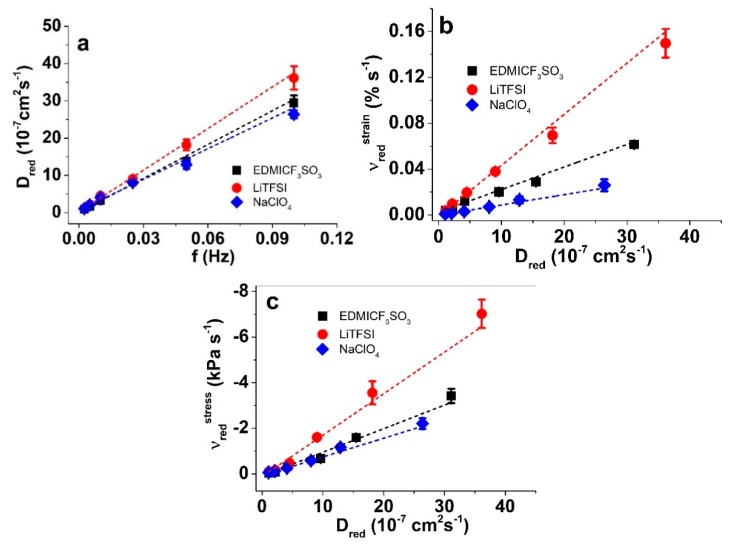
(**a**) diffusion coefficients vs. frequency upon reduction in PP–IPN trilayers in EDMICF_3_SO_3_ (■, black), LiTFSI (●, red) and NaClO_4_ (♦, blue) solutions. The strain rate ν_red_^strain^ in (**b**) and the stress rate ν_red_^stress^ in (**c**) against the diffusion coefficients upon reduction. The dashed lines symbolize the linear fits and are shown as visual guides.

Another view of the matrix–ion interactions is provided by the (apparent) diffusion coefficients, determined using Equations (2) and (3); the results are presented in Figure 6a and Figure S5. The relationship between strain and stress rates and the diffusion coefficients are shown in Figure 6b,c.

The diffusion coefficients upon reduction (Figure 6a) and oxidation (Figure S5) appeared highest in LiTFSI-PC, followed by EDMICF_3_SO_3_ and NaClO_4_. This order does not fully explain the CV current density results, as the onset of cathodic peaks appeared first in EDMICF_3_SO_3_, but as step response processes are much further from equilibrium, some differences were expected. With increasing frequency, the diffusion coefficients increased, as expected [15]. Looking at the strain and stress rates as functions of the diffusion coefficients (Figure 6b,c), the higher charge efficiency of strain in LiTFSI is again demonstrated. Obviously, the strongest solvation and the most stable solvation shell make Li^+^ cations behave as the largest; therefore, their flux in and out of the electrode surfaces creates the most deformation. The solvation of Na^+^ is weaker, and while that of EDMI^+^ is even weaker, it is much larger by itself, and hence, they appear more similar. 

### 3.5. Specific Capacitance 

**Figure 7:**
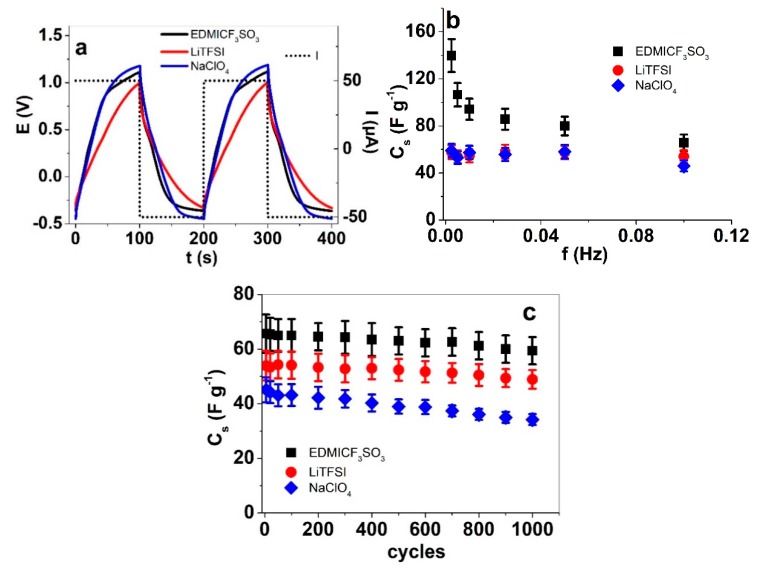
Square wave current steps of PP–IPN trilayers showing in (**a**) the potential time evolution (3rd to 4th cycle) at 0.005 Hz and current ± 50 µA (dotted) and (**b**) the specific capacitance vs. frequency; and (**c**) the specific capacitance vs. cycle number in long term test at 0.1 Hz (± 1 mA). (EDMICF_3_SO_3_ (black, ■), LiTFSI (red, ●) and NaClO_4_ (blue, ♦)).

The mobility of anions and cations in an electroactive polymer material also has an important influence on the charge storage properties. The specific capacitance of a material shows its potential as an energy storage material. To determine the specific capacitance of the PP–IPN trilayers in the three electrolyte solutions, square wave current step measurements were executed from 0.0025 to 0.1 Hz, with currents between ±0.025 to ±1 mA, maintaining a constant charge of 5 mC. The slope used in Equation (4) was determined from the potential time curves of discharging (as shown for 0.005 Hz in Figure 7a) after the IR drop at each applied frequency. The weight of the PP–IPN trilayers was in the range of 72 ± 0.6 µg. Figure 7b shows the calculated specific capacitance (Equation (4)) against the applied frequency; the cycle-to-cycle stability of the specific capacitance of the PP–IPN trilayers at 0.1 Hz are shown in Figure 7c.

If the potential time curves (Figure 7a) are concurrent in the successive cycles, the system is in balance, which means that charging/discharging is in the so called “steady state” [35]. Figure 7b shows the specific capacitance against the applied frequency for PP–IPN trilayers in the three electrolyte solutions. The trilayer operated in EDMICF_3_SO_3_ showed the highest specific capacitance, i.e., 140 F g^−1^ at 0.0025 Hz. Values of 103 F g^−1^ have been shown before for PEDOT electrodes in Et_4_NBF_4_/acetonitrile [9]. Until now, only composites using nanostructured additives such as CNT have reached specific capacitances in the range of 148 F g^−1^ [36]. Apparently, by using ink-jet printing and an appropriate electrolyte, pure PEDOT:PSS is capable of similar capacitances. As is common for electroactive polymers, the specific capacitance in EDMICF_3_SO_3_ decreased with increasing frequency to 65.6 F g^−1^ at 0.1 Hz. However, the trilayers operated in LiTFSI and NaClO_4_ revealed rather odd behavior, as the specific capacitance value remained in the range of 54–56 F g^−1^, virtually independent of the applied frequency and current. This discrepancy can again be explained by the apparent dominant charging mechanisms, which in LiTFSI and NaClO_4_, have more of a capacitive nature following the EDL charging mechanism, while in EDMICF_3_SO_3_, the trilayers behave more like typical conducting polymers, following the redox (Faradaic) mechanism. However, the cycle to cycle stability of the specific capacitance does not depend significantly on the apparent mechanism, as seen in Figure 7c for 0.1 Hz. After 1000 cycles, the specific capacitance of all the PP–IPN trilayers was reduced, almost by a quarter in the worst case, i.e., for NaClO_4_. In the case of EDMICF_3_SO_3_, the specific capacitance of 65 F g^−1^ at cycle 5 decreased to 59.4 F g^−1^ by cycle 1000 (decrease of 8%). For energy storage applications like supercapacitors, PP–IPN trilayers in a PC solution of EDMICF_3_SO_3_ would be most suitable, as they offer the best capacitance and the highest stability, at least after 1000 cycles.

## 4. Conclusions

Ink-jet printed PEDOT:PSS as the electroactive material on both sides of semi-inter-penetrated polymer networks were studied in three different electrolyte solutions, comparing the behavior as well as the underlying electroactivity mechanisms. As an actuator, the printed PEDOT:PSS responded by expansion to discharging (reduction), as the solvated cations enter the film to balance the left-over negative charge of the PSS^−^ anions. Of the three studied electrolytes, EDMICF_3_SO_3_ showed the most redox (Faradaic) character, which was likely related to its high conductivity allowing it to reach to higher potentials in the “bulk” of the electrodes. LiTFSI and NaClO_4_ tended to follow more of a double layer capacitance mechanism. Trilayers in LiTFSI showed the maximum strain and stress in linear actuation, due to the highest electro–chemo–mechanical coupling, a consequence of the strong solvation shell of Li^+^. On the other hand, the most suitable electrolyte in an energy storage application scenario would be EDMICF_3_SO_3_, as the trilayers in that electrolyte showed specific capacitances of up to 140 F g^−1^, as well as the highest stability over 1000 cycles at 0.1 Hz. Finally, ink-jet printed PEDOT:PSS may become a mass-producible electroactive electrode material which would be usable both as an actuator in soft robotics or as an energy storage material, provided the appropriate selection of electrolytes is made, thereby tuning the apparent charging mechanism.

## Figures and Tables

**Table 1:** PP–IPN trilayer electronic conductivities before and after actuation in different electrolytes.

Electrolytes in PC	Before Actuation (S cm^−1^)	After Actuation (S cm^−1^)	Swelling Rate After Actuation (%)
EDMICF_3_SO_3_	104 ± 8	114 ± 9	11 ± 0.8
LiTFSI	87 ± 7	93 ± 8	15 ± 1.1
NaClO_4_	63 ± 4	54 ± 3	7 ± 0.6

**Table 2:** Elastic modulus of PP–IPN trilayers before and after actuation cycles.

PP–IPN Trilayer Electrolytes in PC	Elastic Modulus before Actuation (kPa)	Elastic Modulus after Actuation (kPa)
EDMICF_3_SO_3_	26.7 ± 1.6	98.4 ± 4.8
LiTFSI	17.8 ± 1.1	49.0 ± 3.2
NaClO_4_	22.3 ± 1.1	106.9 ± 5.5

## Supplementary Materials

The following are available online at https://www.mdpi.com/1996-1944/13/2/491/s1, Figure S1: Scheme of actuator fabrication of printed PEDOT:PSS (~2–3 µm) on NBR/PEO membrane (thickness ~7 µm) on both sides forming the PEDOT:PSS-IPN-trilayer (PP-IPN-Trilayer), Figure S2: Square wave potential step response with stress against time of PP-IPN-trilayer at applied frequencies 0.0025 Hz (black line), 0.005 Hz (red line), 0.01 Hz (green line), 0.025 Hz (blue line), 0.05 Hz (cyan line) and 0.1 Hz (pink line) in a: EDMICF3SO3-PC and b: LiTFSI-PC, Figure S3: Square wave potential steps of PP-IPN-trilayer showed in a: current density j curves in EDMICF3SO3 (black line), LiTFSI (red line) and NaClO_4_ (blue line) using propylene carbonate as solvent at applied frequency 0.005 Hz (3rd to 4th cycle) with potential E range at 1.0 to −0.6 V (dotted line) against time t. At different applied frequencies 0.0025 to 0.1 Hz in b: the charge densities Q of EDMICF3SO3 (■ oxidation, □ reduction), LiTFSI (● oxidation, ○ reduction) and NaClO_4_ (♦ oxidation, ◊ reduction) electrolytes in PC against the logarithmic scale of frequency f are shown, Figure S4: Square wave potential steps at applied frequency of 0.1 Hz for PP-IPN-layer showing charge densities Q of EDMICF3SO3 (■), LiTFSI (●) and NaClO4 (♦) against cycle number, Figure S5: Diffusion coefficients at oxidation obtained from Equations (2) and (3) of PP-IPN-trilayer against applied frequencies 0.0025 to 0.1 Hz in electrolytes EDMICF3SO3 (■, black), LiTFSI (●, red) and NaClO4 (♦, blue).
