# Supplementary material for: Printed PEDOT:PSS Trilayer: Mechanism Evaluation and Application in Energy Storage
Source: Materials (Basel). 2020 Jan 20;13(2):491. doi: 10.3390/ma13020491 (PMC7013632; doi:10.3390/ma13020491)
Supplement: Supplementary file 1 [file materials-13-00491-s001.pdf]

materials

MDPI

Supplementary Materials

Printed PEDOT:PSS Trilayer: Mechanism Evaluation and Application in Energy Storage

Inga Põldsalu [1,2], Kätlin Rohtlaid [3], Cedric Plesse [3], Frédéric Vidal [3], Ngoc Tuan Nguyen [4], Anna-Liisa Peikolainen [1], Tarmo Tamm [1] and Rudolf Kiefer [4,*]

[1] Intelligent Materials and Systems Lab, Institute of Technology, University of Tartu, Nooruse 1, 50411 Tartu, Estonia; anna.liisa.peikolainen@ut.ee (A.P.); tarmo.tamm@ut.ee (T.T.)
[2] Centre for Molecular Medicine Norway, Faculty of Medicine, University of Oslo, 0318 Oslo, Norway inga.p6ldsalu@gmail.com (I.P.)
[3] Laboratoire de Physicochimie des Polymères et des Interfaces, Université de Cergy-Pontoise, 15 mail Gay Lussac, 95031 Cergy-Pontoise, France; katlin.rohtlaid@gmail.com (K.R.); cedric.plesse@u-cergy.fr (C.P.); frederic.vidal@u-cergy.fr (F.V.)
[4] Faculty of Applied Sciences, Ton Duc Thang University, Ho Chi Minh City 700000, Vietnam; rudolf.kiefer@tdtu.edu.vn (R.K.), nguyenngoctuan@tdtu.edu.vn (N.N)
* Correspondence: rudolf.kiefer@tdtu.edu.vn; Tel.: +886-90-560-5515

Assembly of PP-IPN trilayer

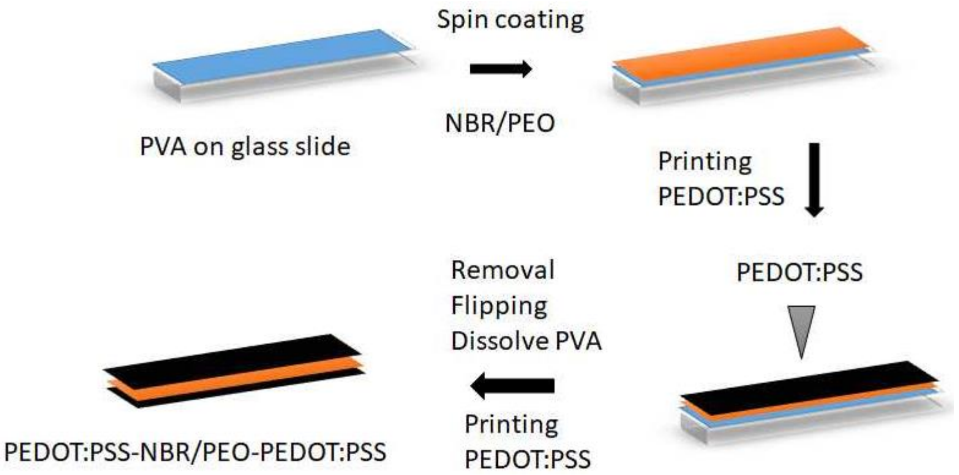

Figure S1. Scheme of actuator fabrication of printed PEDOT:PSS (~2–3 μm) on NBR/PEO membrane (thickness ~7 μm) on both sides forming the PEDOT:PSS-IPN-trilayer (PP-IPN-Trilayer).

In the first step, an aqueous solution (300 g/l mixed at 60 °C overnight in distilled water) of polyvinylalcohol (PVA, Aldrich, Mw = 6000 g mol^{-1}) was spin coated on a glass slide (1,500 rpm/750 rpm s^{-1}/15 s) with additional drying at 80 °C for 5 min. The NBR/PEO solution was spin coated (3,000 rpm / 3,000 rpm s^{-1}/30 s) on the sacrificial PVA layer followed by treatment under the heating bell under argon and curing time of 4 h at 50 °C. DCPD initiator was added NBR/PEO layer to assure complete polymerization of the methacrylate derivate.

On top of the sacrificial PVA layer, the NBR/PEO solution was spin coated (3,000 rpm/3,000 rpm s^{-1}/30 s), followed by treatment under the heating bell under argon and curing time of 4 h at 50 °C for additional polymerization of the methacrylate derivatives using the DCPD initiator. IPN polymer films were obtained in thickness of ~7μm. The next step (Figure S1) contains printing of PEDOT:PSS (electroactive layer) on top of the IPN membranes. PEDOT:PSS (Clevios™ P Jet 700) aqueous solution (solids content 0.6–1.2 wt.%) was purchased from Heraeus Precious Metals GmbH & Co (Germany). To prepare the ink for printing, the aqueous solution was filtrated through a syringe filter (Minisart® NML Syringe Filters 17594-K) with 5 μm pore size to remove larger particles in order to avoid clogging of the print jet. 10 layers of PEDOT:PSS were printed at 800 Hz (customized waveform) on IPN membrane (substrate holder heated up at 45 °C to enhance evaporation of solvent) using a

Jetlab®II Precision Printing Platform with a PH-46 drop-on-demand printhead (MicroFab Ltd., USA.) and a piezo-electrically driven MJ-AT-01 dispensing device with an orifice diameter of 50 μm.

The composite was thereafter additionally dried in a vacuum oven at room temperature at 1 mbar pressure to obtain a solid polymer film and then submerged overnight in a water reservoir with the printed side downwards, facing a Teflon sheet, to allow the dissolution of the PVA layer. The supporting microscope glass was carefully removed, the water was pipetted out to allow the membrane to become flat on the supporting Teflon surface. After drying the sample in vacuum oven at room temperature for 3 h, 10 layers of PEDOT:PSS were printed on the other side of the membrane, aligned with the first electrode layer. The trilayer actuators were cut from the membrane with a scalpel without touching the electrodes to limit the possibility of short circuiting.

Square wave potential step measurements

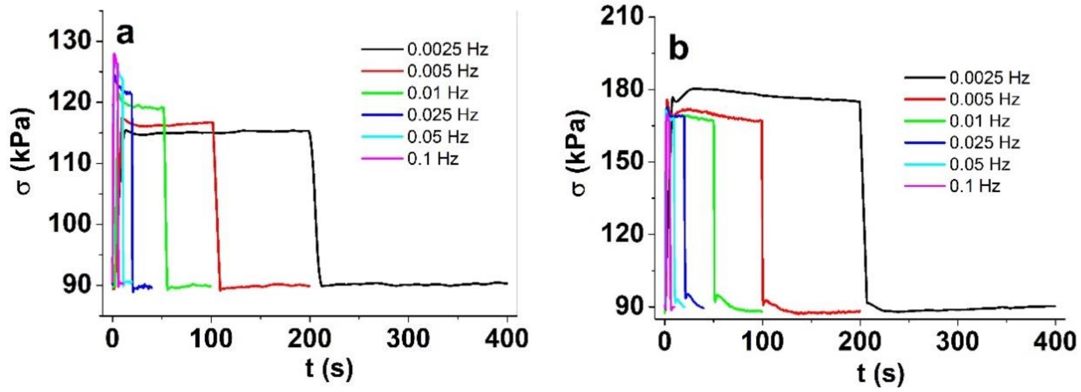

Figure S2. Square wave potential step response with stress against time of PP-IPN-trilayer at applied frequencies 0.0025 Hz (black line), 0.005 Hz (red line), 0.01 Hz (green line), 0.025 Hz (blue line), 0.05 Hz (cyan line) and 0.1 Hz (pink line) in a: EDMICF3SO3-PC and b: LiTFSI-PC.

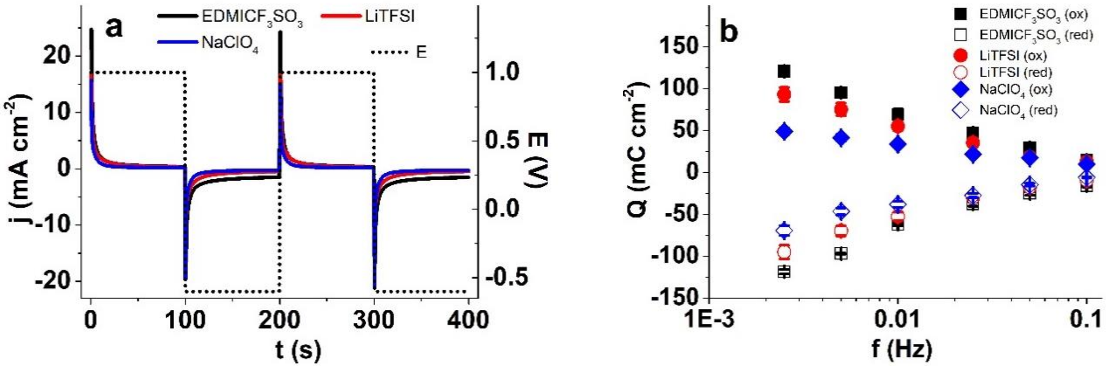

Figure S3. Square wave potential steps of PP-IPN-trilayer showed in a: current density j curves in EDMICF3SO3 (black line), LiTFSI (red line) and NaClO4 (blue line) using propylene carbonate as solvent at applied frequency 0.005 Hz (3rd to 4th cycle) with potential E range at 1.0 to −0.6 V (dotted line) against time t. At different applied frequencies 0.0025 to 0.1 Hz in b: the charge densities Q of EDMICF3SO3 (■ oxidation, □ reduction), LiTFSI (● oxidation, ○ reduction) and NaClO4 (♦ oxidation, ◊ reduction) electrolytes in PC against the logarithmic scale of frequency f are shown.

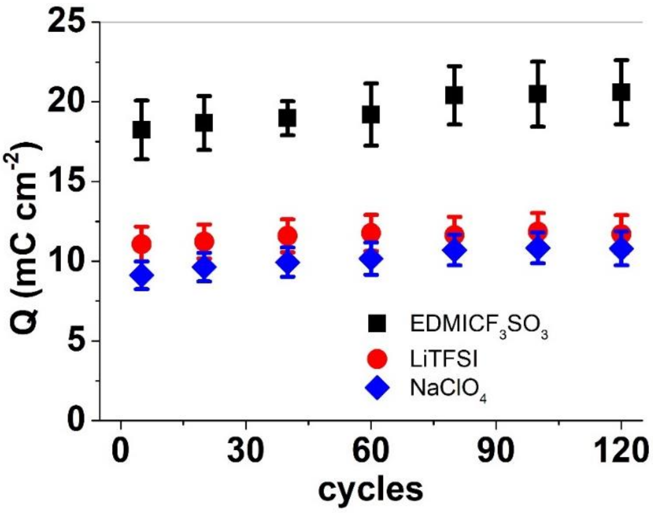

Figure S4. Square wave potential steps at applied frequency of 0.1 Hz for PP-IPN-layer showing charge densities Q of $EDMICF_3SO_3$ (■), LiTFSI (●) and $NaClO_4$ (♦) against cycle number.

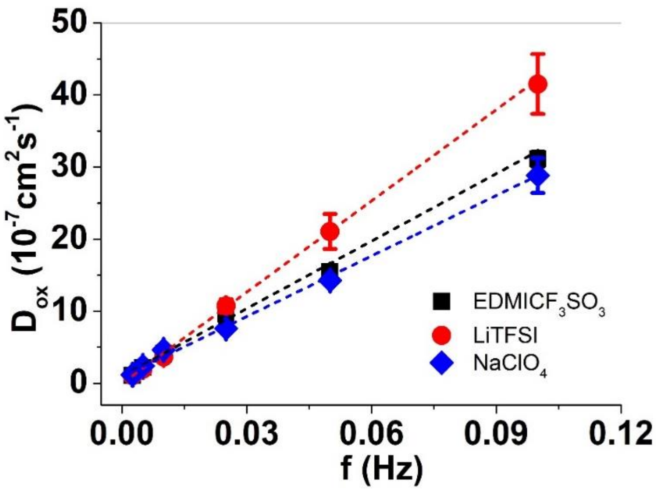

Figure S5. Diffusion coefficients at oxidation obtained from Equations 2 and 3 of PP-IPN-trilayer against applied frequencies 0.0025 to 0.1 Hz in electrolytes EDMICF3SO3 (■, black), LiTFSI (●, red) and $NaClO_4$ (♦, blue).
